# Fossil Carder Bee's Nest from the Hominin Locality of Taung, South Africa

**DOI:** 10.1371/journal.pone.0161198

**Published:** 2016-09-28

**Authors:** Jennifer F. Parker, Philip J. Hopley, Brian F. Kuhn

**Affiliations:** 1 Institute of Archaeology, University College London, London, United Kingdom; 2 Department of Earth and Planetary Sciences, Birkbeck, University of London, London, United Kingdom; 3 Department of Geology, University of Johannesburg, Johannesburg, South Africa; Universidade do Algarve, PORTUGAL

## Abstract

The Buxton-Norlim Limeworks southwest of Taung, South Africa, is renowned for the discovery of the first *Australopithecus africanus* fossil, the ‘Taung Child’. The hominin was recovered from a distinctive pink calcrete that contains an abundance of invertebrate ichnofauna belonging to the *Coprinisphaera* ichnofacies. Here we describe the first fossil bee’s nest, attributed to the ichnogenus *Celliforma*, from the Plio-Pleistocene of Africa. Petrographic examination of a cell lining revealed the preservation of an intricate organic matrix lined with the calcitic casts of numerous plant trichomes–a nesting behaviour unique to the modern-day carder bees (Anthidiini). The presence of *Celliforma* considered alongside several other recorded ichnofossils can be indicative of a dry, savannah environment, in agreement with recent work on the palaeoenvironment of Plio-Pleistocene southern Africa. Moreover, the occurrence of ground-nesting bees provides further evidence that the pink calcrete deposits are of pedogenic origin, rather than speleogenic origin as has previously been assumed. This study demonstrates the potential value of insect trace fossils as palaeoenvironmental indicators.

## Introduction

The discovery of the first *Australopithecus* fossil, the juvenile ‘Taung Child’ at the Buxton-Norlim limeworks [1], was followed a decade later by discoveries of adult gracile and robust australopithecines from Sterkfontein and Kromdraai [2, 3]. Ninety years on and the expanse of southern Africa has yielded an impressive collection of palaeoanthropological finds. Despite excavations in the 1940s, 50s, and 90s, no further australopithecine fossils have been unearthed at Taung. In recent years, the focus of research at the site has shifted to the taphonomic, environmental and geological context of this hominin locality, as well as some of the other known fossil deposits.

The nest described here was excavated from the Type Site at the Buxton-Norlim Limeworks in South Africa, which lie in the Ghaap Escarpment at the south eastern edge of the Kalahari. The limeworks contain a large system of tufa deposits that are located approximately 15km south west of the village of Taung. Just north of the presumed discovery site of the ‘Taung Child’, two pinnacles were left as witness sections; these are referred to as the ‘Dart Pinnacle’ to the west and the ‘Hrdlička Pinnacle’ to the east [4].

Two lithologies have been identified throughout the Dart and Hrdlička Pinnacles [5]. These are: pink clay and siltstone (PCS) and a yellowish-red sand and siltstone (YRSS) [6]. The analysis of these lithologies revealed that the PCS deposits at the base of the Dart Pinnacle closely match the matrix most often associated with the ‘Taung Child’ [6]. It is thus understood that the PCS deposits are remains of the same deposits from which the ‘Taung Child’ cranium was recovered [4]. It was from these PCS deposits at the Type Site that the fossil bee’s nest was recovered in 2010.

Although typically described as cave sediments (e.g. [7, 8]), a recent sedimentological analysis implies that the deposits may in fact be of pedogenic origin [9]. The pink calcrete deposits are likely to have formed on the land surface and the sediment consists principally of micrite (microcrystalline calcite), but also contains sparry calcite cement and silt-sized quartz grains [9]. A range of sedimentological features common to large calcretes is present in the Type Site PCS. Hopley and colleagues (2013) recorded the presence of rhizoconcretions, root mats, and trace fossils within the deposits [9], all of which are suggestive of paleosol development. The PCS has been subject to phases of permeation over millions of years resulting in the cementation of carbonate, and in turn, excellent preservation of fossils.

There is very little ecological or environmental information available for the Taung locality to date, and even less specific to the Type Site. The majority of palaeoenvironmental assumptions following analysis of the ‘Taung Child’ were based on faunal remains and sediment analyses. A number of small- to medium-sized animals have been identified at the site, all of which are well adapted to living in cave, rocky, or edaphic microhabitats [10]. This accumulation contrasts with the general Transvaal faunal assemblage, which includes a great sample of large, mobile mammals [10]. It should be noted that the vast majority of faunal remains were found in YRSS deposits of the Hrdlička Pinnacle; very few have been published from the PCS deposits that contained the australopithecine child [10, 11].

This paper will attempt to identify the inhabitants of a bee’s nest recovered from the PCS of the Type Site at Taung using the literature on classification that is available and comparison with other fossil bees’ nests that have been described in publications. The presence of the ichnospecies and ichnofacies identified at the site will be used in conjunction with other published data to evaluate the likely palaeoecology and palaeoenvironment of Taung Type Site in the Plio-Pleistocene. Finally, any implications that the presence of a species of ground-nesting bee may have on the interpretation of the Type Site deposits will be explored.

### Bees and their Nests

The majority of modern-day bee species nest in the ground and are known to construct their nests in a diverse range of soil types including (but by no means limited to) sandstone, clay, alluvial silts, desert sand dunes, and beaches [12]. The cells in which the larvae grow are only thinly lined and so in more humid regions, the provisions placed in the cells are likely to be attacked by fungi; a humid environment may also result in the liquification of provisions, which could cause the immature bees to drown [13]. Typically, the species that are more successful in humid regions are those that do not nest in the ground [13].

Bees’ nests can often be found in clusters; this is common due to the fact that solitary bees search for sites with specific qualities [12]. Nests are constructed by the females and generally consist of a main burrow in the ground which gives rise to several lateral burrows, each of which terminates in a chamber, or cell [12]. These cells usually contain a single larva and can be provisioned with pollen, nectar, and other plant material [12]. The cells may be lined or unlined, and the burrows are never lined; typically, each lateral burrow is filled with the debris from the creation of the next [13].

Although dependent upon the species, the majority of these nest makers line their cells with an earthern layer, such as a fine clay; they then smooth the surface with the pygidial plate, and apply a cellophane- or wax-like (it does not actually contain any wax) substance that is secreted by the bee [13]. Both the layer of finer earth and the secreted lining are derived features that are unique to bees [13]. The insoluble substance often used to line larval cells is secreted primarily from the Dufour’s gland, which is located at the base of the bee’s sting and is usually large in ground-nesting bees [13]. The secretion forms a relatively watertight membrane around the larvae, allowing it to withstand varying environmental conditions [12]. It is in these cells that the larvae spend most of their lives before emerging as adults [12, 14].

### Fossil Bees’ Nests

Although fossils of bees are rare due to their delicate body structures [13], the traces of bees can be found in much less specific conditions. Bees’ nests are intricate structures that are regularly found in paleosol deposits. The first fossil bees’ nests were described over a century ago [15, 16]. It wasn’t until the 1930s however, that bee cells were formally identified and named. It was at around this time that Brown (1934, 1935) erected the ichnogenus *Celliforma* that, at the time, included all fossil bees’ nests recorded to date [17, 18]. The original description of *Celliforma* proposed by Brown (1935) included “all fossil fillings of chambers purporting to have been made originally by unknown mining Hymenoptera” [18]. It was not until 2000 that a new, more specific system of classification was proposed [19].

Only one other fossilised bees’ nest has been recorded from present day Africa. Thackray (1994) described a nest of sweat bees from Rusinga Island, Western Kenya, which dated back to the Miocene [20]. This nest was attributed to the ichnogenus *Celliforma*, but, in accordance with recent refinements to the classification [19], it would no longer belong to this group due to the fact that the cells are not solitary. The cells within this nest are arranged in a very specific, paired pattern, allowing for its interpretation as a nest of social bees. The presence of this nest provided an indication of the palaeoecology of Early Miocene Kenya, identifying it as an area dominated by angiosperms with bare, relatively dry soil in a subhumid climate [20].

The identification of insect trace fossils and ichnofacies can prove extremely useful to studies of palaeoecology and palaeoenvironment. Insects often incorporate organic matter in to their nests and, in consequence, are enormously important to the process of soil formation; an insect nest can be a very reliable sign of soil development and thus can provide clear evidence of the presence of paleosols [21]. Sensitivity to local environmental factors including soil condition, microclimate, and vegetation, means that insects tend to nest in very specific conditions [21]. In terms of hymenopteran insects, if too much moisture enters the larval cells then fungi and other organisms will attack provisions, if on the other hand there is not enough moisture, the larvae will suffer from dehydration [21].

The identification of cell linings in fossilised bee cells can also provide important information with regards to the local environment. Cell linings can provide a defence against unfavourable conditions and might be incorporated to prevent water from entering the cells [12]. Few groups of bees nest in humid conditions; those that are successful in such conditions rarely nest in the soil, but there are some that do, and these typically excavate complex cells, for example, using thick resinous materials to line and seal the cells (Michener, 1978). Cells made without linings on the other hand might be indicative of a more favourable environment for the larvae.

As the microclimate and vegetation are dependent on the climate as a whole, the nests can provide valuable information about the palaeoclimate and palaeoecological conditions [21]. Insects regularly produce recognisable traces throughout the world and this is a feature that could be exploited to a much greater extent in archaeological and palaeontological studies to expand understanding of palaeoenvironmental conditions.

## Methods

South African Heritage Resources Agency permit number 80/09/10/028/51 was used for the export of geological specimens from Taung. These specimens include: TDTF 1, 2, 2A, 2B, 2C, 3, 4, 5, 5A, 6A, 6B, 6C, and 6D. The specimens belong to the permanent university museum repository at the Fossil and Rock Collection of the University of the Witwatersrand, Johannesburg, Evolutionary Studies Institute, PO Wits, 2050 Wits, South Africa.

The fossil bees’ nest described by Thackray (1994) was prepared by sawing 14 parallel cuts through the nest and recording the 3-dimensional pattern of cells and tunnels [20]. Rather than employing a destructive serial sectioning method, the internal structure of this nest was imaged using micro-CT (computed tomography) facilities at the Natural History Museum, London.

The CT scans provided a series of 2D slices that were transformed into a 3D digital model using Drishti software [22]. Experimentation revealed that the most effective method to display the structure of the inside of the nest pieces was by using the ‘shrink-wrap’ feature offered in Drishti. By ‘shrink-wrapping’ the digital model it was possible to make visible only the areas at which density met air. This provides an image of the outline of any porosity within the matrix. It should be noted that it later became clear that some of the cells were in-filled with sediment, and so not all of the cells present would have been visible in the CT scans.

The nest and visible individual cells were measured, and their shapes, orientation and pattern recorded in order to provide an overall description of the nest as a whole. It proved difficult to determine the proximal from distal ends of the cells that were still encased in the matrix. There were also only a small number (25) of visible cells on the dorsal surface of the nest, making it difficult to observe the density and pattern of cell distribution within the calcrete blocks.

A visible cell from the underside of block 1 that had been broken in situ was thin sectioned for petrographic analysis. The section was prepared at Birkbeck College’s Department of Earth Sciences, and then viewed with a petrographic microscope under plain polarised light. Photographs were taken to document the structure of the cell lining. The sample was also viewed under a confocal microscope at the UCL Confocal Microscopy Unit, with the use of Leica Lite software in an attempt to see whether or not the trichomes within the lining emitted fluorescence as an organic structure.

## Results

**Fig 1:**
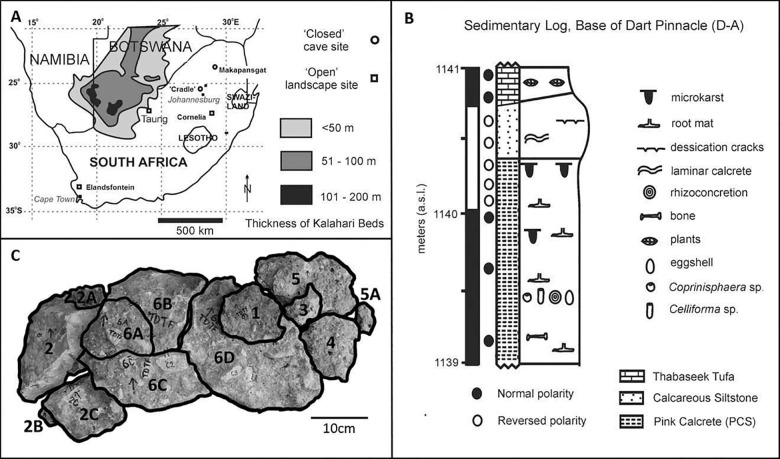
Locality and Stratigraphy of the Deposits. (A) Locality of the Buxton Limeworks, Taung, South Africa (from Hopley et al. 2013). (B) Stratigraphy of the trench excavated at the base of the Dart Pinnacle, locality D-A (from Hopley et al. 2013). (C) Photograph of the fossil bees nest collected from the Pink Calcrete (PCS), exposed in locality D-A at a height of 1140.5 m (a.s.l.). Each block has been numbered for reference purposes.

**Fig 2:**
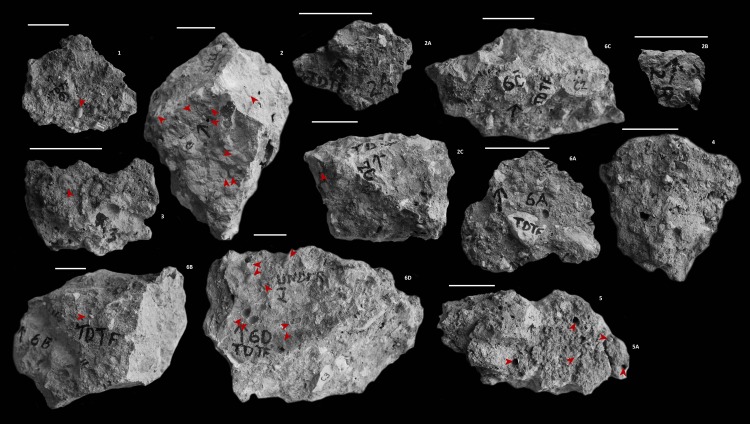
Photographs of each of the Individual Pieces of Extracted Nest. The numbers are at the top right of the pieces, and the scales (5cm) are above the pieces that they are pertaining to. Arrows highlight visible cells in the deposits.

The whole specimen is approximately 115cm in length and 50cm in height when pieced together (see Fig 1C). There are 25 visible cells on the outer surface of the nest. Fig 2 highlights the cells that are visible in upper view of each of the specimen pieces. Each cell is approximately 14mm in length and approximately 7mm at the widest point. There is no particular pattern to the cell arrangement; they do not appear to be arranged in clusters or in rows.

**Fig 3:**
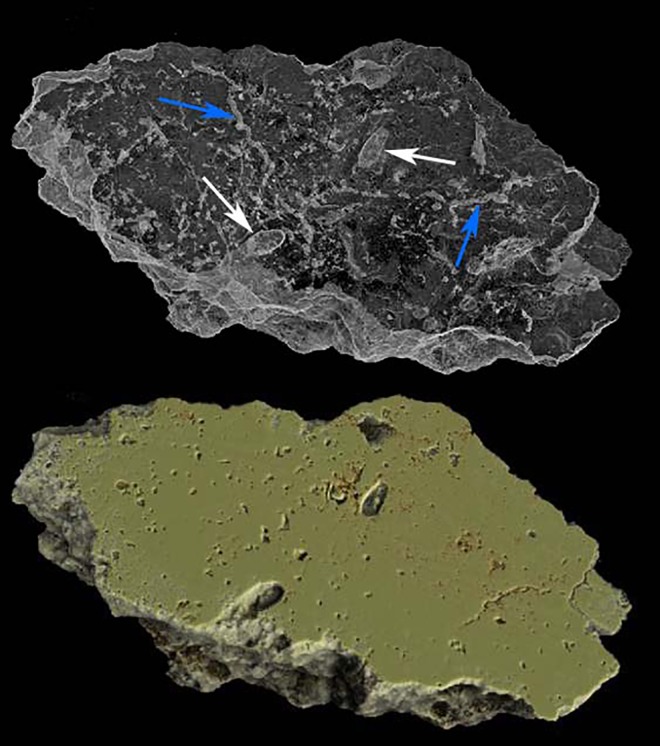
CT Images of slice of Block 5 in shrink-wrapped form (above) and solid form (below). Note the complex matrix of porosity, *Celliforma* cells (white arrows) and tunnels made by unknown organism (blue arrows).

**Fig 4:**
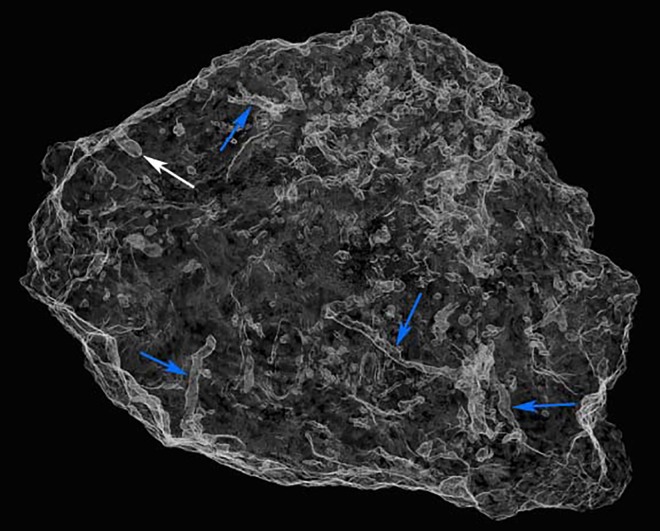
CT Image of Block 6D in shrink-wrapped form. Note the complex matrix of porosity, *Celliforma* cell (white arrow) and tunnels made by unknown organism (blue arrows).

A few cells can be confidently identified from within the sediment blocks using micro-CT (e.g. Fig 3), but the partial or total filling of the cells with sediment makes it very difficult to state with certainty how many additional cells are present inside the blocks. The images do however reveal an unexpected network of primary porosity within the blocks (a secondary porosity formed by carbonate dissolution is unlikely to have such an intricate shape). The dense matrix of differing shapes in the porosity can be attributed to living organisms–be these plants or insects. Of particular interest are several tunnel-like structures (highlighted in Fig 4) identifiable within the matrix. These tunnels are not likely to be related to the bee trace maker as the tunnels of bees are never lined and are usually filled in with sediment during the nest construction [13]; the tunnels also do not appear to terminate in a cell. As with the majority of porosity in the specimens, the tunnels are indicative of a wider ichnofauna–perhaps representing the traces of a different species of insect, or the root traces of a plant.

**Fig 5:**
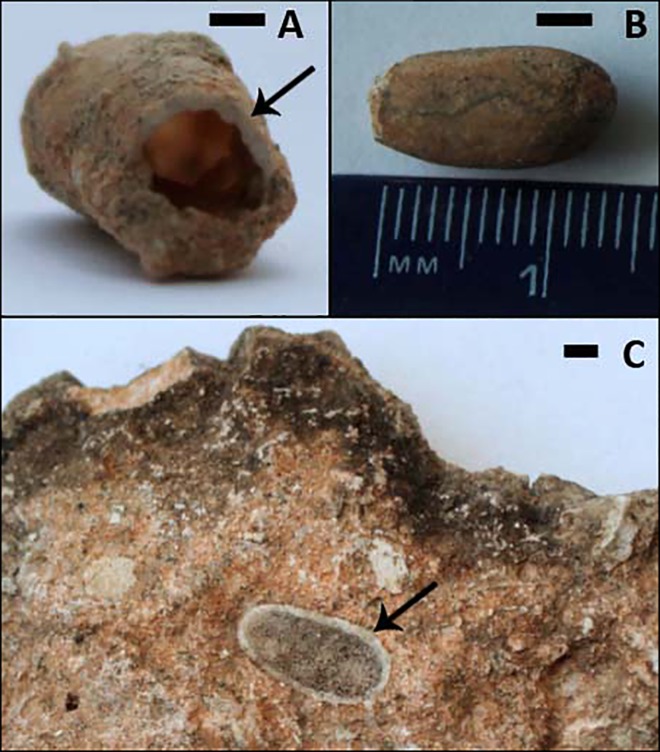
Three Different Individual Cells. (A) and (B) have been extracted from the nest, and (C) (although broken in half laterally) remains in the matrix. (A) displays a proximal view of a cell that has been broken at the neck (the entrance); note the cell lining (arrow). (B) displays a dorsal view of a cell, note the smooth cell wall; the proximal end (at the left of the image) has been broken at the neck. (C) shows a dorsal view of a cell that has been broken laterally; the calcite lining (arrow) is clear, as is the cell shape (narrower and blunter at the proximal (left) end, more rounded at the distal (right)). Scale bar = 3mm.

The individual bee cells are flask-shaped (with the inclusion of the unlined entrance burrow). The main compartment is oval-shaped with very smooth, polished walls and a distinctive calcite lining of approximately 1mm in width (see Fig 5); the cell then narrows at the neck, and expands where the unlined entrance burrow can be seen at the proximal end. Most cells are hollow, but a few appear to be in-filled with sediment. The maximum diameter of around 7mm is reached approximately one third of the way down the mould from the distal end. It is the distal end that is rounded, and the proximal end that is generally truncated where it is broken irregularly at the narrowest point. The cells have a slight curvature on one side (the long axis) (Fig 5). It is possible to make out an entrance burrow in the sediment for most of the cells in the matrix, which is generally a small indent (approximately 5mm long) before the proximal end of the cell begins. The entrance burrows are typically filled with sediment, and often remain intact when individual cells are removed from the matrix. There is no evidence of a spiral cap, or indeed any form of closure in the cells; this is not surprising since closures are not usually preserved in fossil specimens [23].

Petrographic observations indicate that the organic cell lining has been replaced by calcite, but that relics of the organic lining have been preserved. Calcite replacement has occurred at an early stage of diagenesis, as indicated by the preservation of both the organic lining and calcitic casts of plant hairs (trichomes). There is no evidence for in-filling of the cells by a drusy calcite cement, indicating that the cell linings are of a similar thickness and morphology to that constructed by the bee.

**Fig 6:**
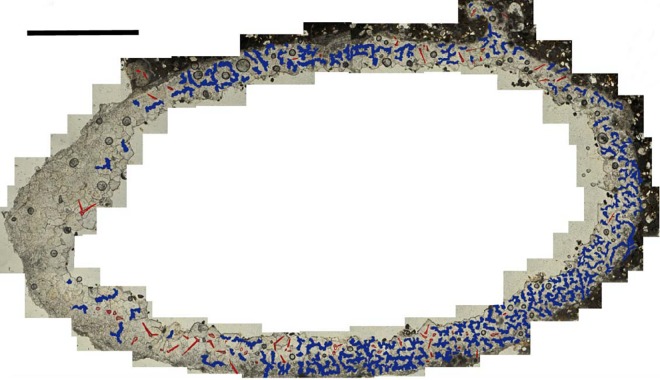
Petrographic Microscope Image of a thin section of a Cell. The calcitic casts of the trichomes are highlighted in red and the relics of an organic matrix in blue. Scale bar = 1.5mm.

**Fig 7:**
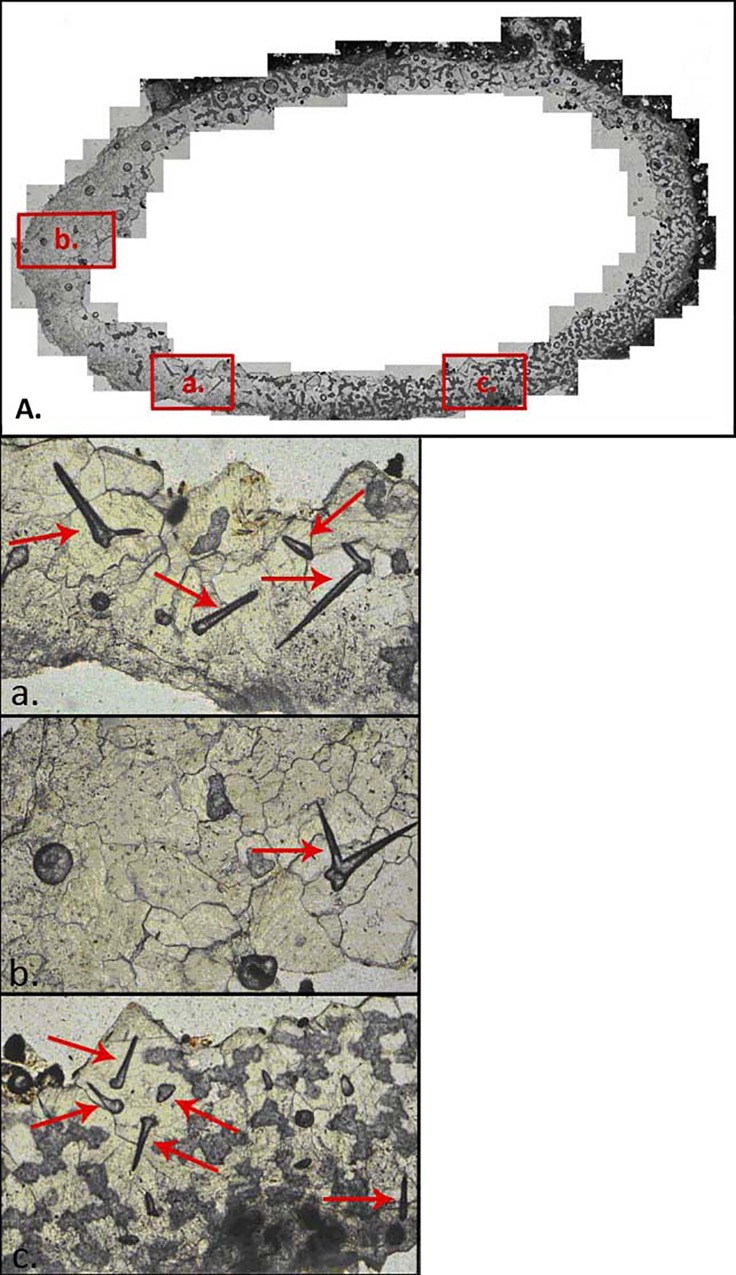
Petrographic Microscope Images of sections of a thin section of a Cell. (A) shows the position of images (a), (b), and (c) in the whole thin section. (a), (b), and (c) display sections of the cell lining at a higher magnification to show the trichomes (arrows) and organic lining structure (c; darker grey). (a), (b), and (c) are 1.5mm in width (left to right).

Inside the cell lining are a large number (around 55) of distinctive hair-like structures (see Figs 6 and 7). These structures are approximately 160 to 350 μm in length and are randomly orientated. Some of these structures appear to be bifurcated from near to the base whilst others do not appear to fork at all. Based on a review of nesting habits of bees, and comparison with images in the literature (see e.g. Gutiérrez-Alcalá et al. 2005 [24]), the structures have been identified as non-glandular plant trichomes. Viewing the sample under a confocal microscope revealed blank fluorescence emission, illustrating that the sample was not excited by UV light and did not contain organic compounds. It can thus be assumed that the trichome structures are calcite replacements of the original structures formed at a very early stage of diagenesis.

## Discussion

### Ichnotaxonomy

The smooth, polished walls of the cells and the organic features of the lining allow for the attribution of the nest to a bee, rather than a wasp [25]. The cells are not attached to a tunnel or arranged in clusters of adjacent rows, so can be classified as solitary bee cells. It has been assumed that the nest was made by a single bee; members of Megachile have been reported to lay up to 35 eggs in favourable conditions [26]. Alternatively, it may be a cluster of nests made by several bees; solitary bees are known to nest in clusters in areas where conditions are ideal [12].

**Fig 8:**
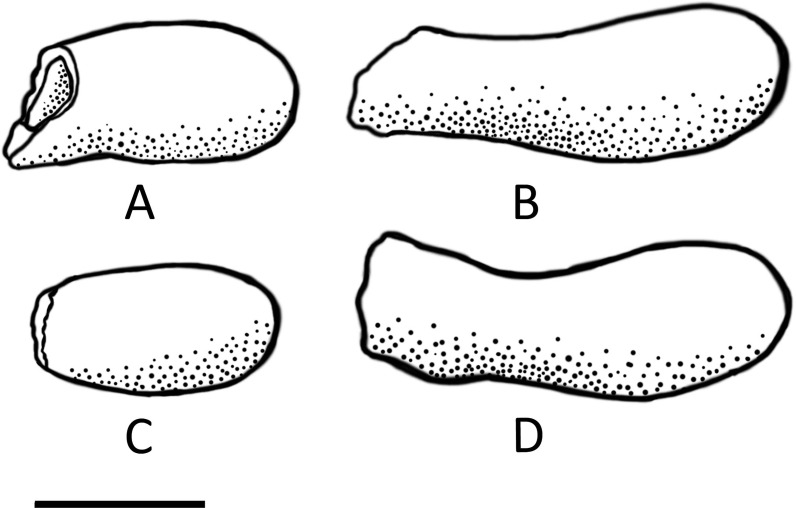
Illustration of Internal Moulds of four different cells extracted from the Fossil Nest, preserved in varying degrees of completeness. The shape of some exceptionally preserved, almost complete cells (B, D) can be seen (with the inclusion of antechambers), as well as incomplete cells–the way that the majority are preserved, having broken off at the narrowest point, where the lining ends (A, C). The straighter long axis (above) and the more curved long axis (below) can be seen in side view of the more complete specimens (B, D). Scale bar = 1cm.

According to the classification provided by Genise (2000), only two ichnogenera have been described for solitary bee cells: *Palmiraichnus* and *Celliforma* [19]. *Palmiraichnus* have an internal structure composed of an ovoid chamber and a spiral cap and a subcylindrical antechamber surrounded by a discrete wall [27]. In contrast, *Celliforma* lacks antechambers or constructed walls [19] and can exhibit a range of different shapes (subcylindrical, tear, flask, urn, vase, or barrel). On the basis of the observations made above (and Figs 5 and 8) it is clear that the Taung Type Site bee cells can be assigned to the ichnogenus *Celliforma*. This nest represents the first recording of *Celliforma* in Africa, discounting the nest recorded from Rusinga Island (western Kenya) [20] that, based on recent amendments to the classification system [22], would no longer belong in this ichnogenus. It also represents the second fossilised bee’s nest of any classification to be described from Africa.

The remnants of an organic substance are clear in the petrographic microscope images of the thin section. The structure (highlighted in blue in Fig 6) forms a very intricate pattern and, as with the trichomes, is absent from the proximal portion of the cell. Although a few papers have described microscope images of thin sections of fossil bee cells in the past, only one has mentioned the possible presence of an organic lining. La Roche et al. (2014) described a fossil bees’ nest from the Canary Islands [28]. Mentioned in the paper is the presence of an organic lining within a thin section. This structure is not as well defined as that being described here, but nevertheless it is possible to see some similarity between the two.

The current study provides the first report of trichomes observed in the lining of a fossil bee cell. The trichomes lack the globular structure involved in the secretion of phytochemicals that appears to be distinctive of glandular trichomes; they are thus classified as non-glandular trichomes. Non-glandular trichomes are specialised structures derived from the epidermal layer of the plant; they function primarily as physical defensive structures against herbivores [24], but also aid the absorption of water [29] and act as a sink for toxic heavy metals and xenobiotics [30].

The even distribution of the trichomes throughout the cell lining implies that they were added to the lining by the nest-maker purposefully. Note also that they are absent from the proximal portion of the cell lining–most likely because this is where the larvae destroyed the lining as it emerged from the cell. Exactly how the bee would have extracted the trichomes from the plants is unclear. Some species of modern day carder bee have adapted mandibles for use specifically to dismantle trichomes [13]. These trace makers may have used a similar mechanism, though without body fossils it will not be possible to state with certainty what mechanism was used. Interestingly, the trichomes present in the lining of this cell do not appear to have been sharply cut, sheared, or chewed from the plant.

Although it is possible that the trichomes were removed as an entity, another possibility is that the bee lined the cells with leaves and that the trichomes were the only structures that fossilised. If the bee was lining the cells with neatly cut pieces of leaf, a preferred orientation might be expected. The random orientation of the trichomes could imply that the bee formed a pulp of leaf material combined with a secretion (perhaps resin, or a secretion obtained from the leaves or stem of a plant)–a behaviour that is seen in some species of Megachile today [13]. This would explain the presence of both the trichomes and the organic substance that was applied to the walls. The calcitic replacement of this organic secretion is revealed as a complex matrix (highlighted in blue in Fig 6).

The presence of trichomes in the lining of the cells is a feature that has not been recorded in a fossilised nest before. It must be mentioned however, that the presence of this feature is probably the result of exceptional preservation in these specimens. The majority of other nests described in the literature were not preserved to the same degree and so should trichomes originally have been present, poorer preservation would mean that they might not be visible in the fossils. These fossils may represent a new ichnospecies, but at present (until a more precise taxonomic classification is available), it seems best to classify the structures at an ichnogeneric level. The nest most closely resembles those of *Celliforma*, containing solitary cells that are rounded at one end and truncated at the other, with smooth walls and no clear antechamber.

No other *Celliforma* nests have been recorded from Africa, and the nest cannot be placed into the criteria of any of the described ichnospecies within *Celliforma* from other parts of the world with any level of confidence. The ichnospecies-level classification is very complex and, at present, problematic [19]. A number of trace fossils have been attributed to *Celliforma* but have not been named; the only named ichnospecies still believed to belong to the ichnogenus appear to be: *Celliforma spirifer* (Late Eocene, USA) [17], *C*. *nuda* (Early Miocene, USA) [17], and *C*. *germanica* (Oligocene, Germany) [18]. *C*. *nuda* and *C*. *spirifer* are both named after the distinct spiral closures of the cells–a feature that has not been identified in these cells. The type specimen for *C*. *spirifer* also averaged 270mm in length, making it considerably larger than the cells described here, whilst the cells of *C*. *germanica* are distinctly tear-shaped.

### Comparison with Modern Bee Taxa

The structure of the cells themselves and the components of the cell lining in particular provide some features that are comparable with cells produced by extant species of bee. Ground nesting bees are prevalent throughout the world in the modern day, and different species and genera vary greatly in aspects of their nesting behaviour [13]. A number of large families have ground-nesting members that create subterranean cells like those described here; these include: Colletidae, Andrenidae, Halictidae, Melittidae, Megachilidae, and Anthophoridae [13]. In common with the fossil bee cells found at the Taung Type Site, bee nests in soil are typically comprised of flask-shaped cells, where the narrowest point is the entrance. Groups of bees with members that create this form of cell include Halictidae, Anthophoridae, and Andrenidae [13, 14].

Most bees line their cells with a waxy substance that is excreted from the body of the female bee. It is possible that the organic matrix that is visible in the images of the bee cell extracted from the nest being described here represents the remains of this substance. This lining is usually secreted from the Dufour’s gland with the purpose of maintaining an optimal moisture balance within the cells. However; assuming that the hair-like structures in the lining of the cell examined are indeed trichomes, it is possible to associate the trace makers with extant Megachilidae and more specifically, the sub-family Megachilinae.

The Megachilinae are fairly unique amongst bees in that almost all of them utilise foreign material in the creation of the cells or partitions in their nests. There are only a few other tribes, each of which belongs to Apinae, that are known to incorporate foreign material in their nest construction [13]. The large sub-family includes solitary or cleptoparasitic bees that form nests consisting of cells (or occasionally only partitions of unlined burrows) [13]. The foreign materials are generally obtained from plants and can include cut leaves, chewed leaf pulp, resin, and mud.

A large proportion of megachilids do not excavate their own burrows from scratch. Many adapt plant stems, hollow wood, or nests previously made by wasps, by creating additional burrows, making cavities smaller, and constructing a cell lining made of foreign material [13]. Those that do nest in the open construct roughly cylindrical cells that are irregularly placed in a group [13]. The cell construction in the nest from the Taung Type Site is not consistent with those made by the majority of extant members of Megachilidae. However, some comparisons can be drawn to the nesting behaviours of Megachilinae tribes.

With the exception of Lithurgini, the vast majority of Megachilinae use foreign materials from outside of their nests when creating cells for their larvae [13]. The tribes of particular interest here are those that specifically incorporate plant material; these include: Osmiini, Anthidiini, and Megachilini. Members of Osmiini create nests in stems, in wood, under rocks or in the ground. They might use a ‘gummy leaf pulp’ (consisting of leaf pulp and nectar, resin, or gum) or resin to partition or line the larval cells [13].

Bees that are best known for the inclusion of plant hairs (trichomes) in their nests belong to the tribe Anthidiini–a group more commonly known as wool carder bees that encompasses several genera and over 70 species [13]. Although there is only one known species in Australia, all other continents are home to numerous anthidiine genera and species in the present day [13]. Anthidiine females of the genera *Trachusa* and *Paranthidium* create their own burrows using foreign material such as resin, which is occasionally mixed with soil particles, pieces of leaf, or plant hairs, amongst other materials [13]. Others use plant hairs and fibres alone, sometimes moistening them with glandular secretions from plant leaves or stems to make them easier to manipulate [31].

Finally, bees within the tribe Megachilini are understood to incorporate leaf pieces into their nests, which are usually found in cavities, stems, manmade objects, or burrows in the ground. Most notably, members of the genus *Megachile* have been reported to use leaf pieces, sometimes alongside chewed leaf pulp, to structure their nests, some of which are dug by the bees as burrows in the ground [13].

It has been shown that the use of foreign material to line larval cells creates a hydrophobic membrane and can display antimicrobial properties [32, 33]; it therefore serves a similar purpose to the substrate that is secreted from the body by other families of bee. Interestingly, although many possess a large Dufour’s gland, members of the Megachilidae do not use this secretion for lining their cells; rather, the products of this gland (such as fatty acids and triglycerides) are purported to be used for added nutrition in the larval food [34]. It therefore seems likely that the substance forming the matrix in the cells described here (visible in Fig 6) is plant secretion, resin, or chewed leaf pulp–not the product of Dufour’s gland.

Exactly when in the evolutionary history of Hymenoptera the use of foreign materials to line nests originated is not clear, but evidence of leaf-cutting behaviour in bees (*Megachile*) has been recorded in the form of body fossils dating back to the Paleocene [35, 36]. A review of the literature reveals no other evidence of bees lining their nests with trichomes in fossilised specimens [37].

### Palaeoecological Significance

The conditions in which solitary bees form their nests are relatively well understood. The nest of any bee species must be within proximity to angiosperms that provide the necessary pollen for the bees [37]. Nests are usually constructed on bare, light, dry soil with exposure to the sun [25]; there are however exceptions to this rule [38] and Michener (1964) has also noted that the construction of nests does require a substrate with some moisture, in order to aid soil packing and ensure that the nest does not collapse [39].

*Coprinisphaera* is the name given to an ichnofacies that was first described by Genise and colleagues (2000) [21]. This ichnofacies consists of the trace fossils of various insects, but primarily bees, ants and beetles [21]–all of which have been identified in the PCS deposits of the Taung Type Site (see Hopley et al. 2013 [9]). Termite traces can also be found where the palaeoenvironment was relatively dry, but these have not been discovered at the Taung Type Site. Several components of the *Coprinisphaera* ichnofacies have been identified at other early hominin sites in Africa. Examples include: the aforementioned site on Rusinga Island, where a fossil bees’ nest was recorded [20], a series of fossilised termite nests in Chad, discovered alongside dung beetle traces [40], and finally in Tanzania, where traces of beetles (Coleoptera) were identified [41].

Unlike at other hominin sites, excavations at the Taung Type Site have revealed all of the key ichnofamilies that signify the presence of the *Coprinisphaera* ichnofacies. The occurrence of the *Coprinisphaera* ichnofacies has a moderate to high ichnodiversity and high abundance; it is especially common in mature paleosols, and is generally indicative of savannah grasslands [21]. That dung beetle traces have been identified in the deposits [9] shows that the area is likely to have been herbaceous, such as savannah, grassland or prairie [42]. This is evident as dung beetles are known to provision their nests with the excrement of vertebrate herbivores [21].

The presence of both a bee’s nest and the *Coprinisphaera* ichnofacies are suggestive of a terrestrial herbaceous environment with arid soils, plant growth, exposure to the sun, and the presence of angiosperms [27]. It is most likely that these angiosperms would have been C3 in nature, although pollination of C_4_ grasses is also a possibility [43, 44]. Megachilidae is described as a generally oligolectic family [45]. Anthidiini is a tribe with particularly varied pollen selection between species; 43% of extant species within Anthidiini are narrowly oligolectic, 18% are moderately polylectic, 35% are strongly polylectic, and 4% are unknown [31]. Pollen from the families Fabaceae and Lamiaceae (C_3_ forbs) are however the most favoured amongst members of Anthidiini, after composites [31]. A potentially mixed source of pollen is consistent with carbon isotope analysis of fossil eggshells from the PCS Calcrete at the Taung Type Site [46] which indicates the presence of both C3 (trees, bushes or forbs) and C4 (tropical grasses or sedges) plants.

The trace fossils of ground-nesting insects are an extremely reliable indicator of the presence of paleosols [21]. In most ground-nesting bee species, an egg is laid in each cell, provisioned with a ball of pollen or nectar, and then the adult leaves the cell and seals the entrance with a cap [13]. The larvae inside the cells of solitary insects are usually immobile, and so, unlike social insects such as termites, their nests cannot be reconstructed or moved [21]. The presence of rhizoconcretions, *Coprinisphaera* [9], and eggshell fragments [46] belonging to a ground-nesting bird have been described from the PCS deposit–all of which are indications of paleosol development.

The presence of ground-nesting bee traces provides additional evidence that the PCS deposits are of pedogenic origin. This finding has a number of wider implications for research at the Taung site. Under the cave model of the Dart and Hrdlička pinnacles, it was unlikely that many more in-situ fossils remained to be uncovered. The discovery that the hominin-bearing PCS deposits are instead of pedogenic origin has entirely different implications for future research. Hopley and colleagues (2013) found that the calcrete extends hundreds of metres beyond the pinnacles in the realms of a carbonate-rich river system [9]. This greatly increases the likelihood of more primate fossils being uncovered at the Taung Type Site in future excavations.

### Conclusions

Here the first fossil bee’s nest from South Africa has been described and the trace makers attributed to the ichnogenus *Celliforma*. The results of the study largely reinforce the recent literature regarding the palaeoecology of the Taung Type Site, and indeed of Plio-Pleistocene southern Africa as a whole. Moreover, they strengthen the hypothesis that the Type Site deposits are pedogenic in origin, having formed on the land surface, rather than as sedimentary in-fills of caves as is the case for the majority of southern African hominin sites. Despite recent advancements in the field, the identification and implications of the presence of insect trace fossils in terrestrial deposits remains largely overlooked, especially in early hominin sites. Although common to paleosol deposits, insect traces are rarely considered in detail, yet they could offer important palaeoenvironmental insights, with the potential to reveal valuable information about hominin palaeoecology.
